# Nutrition-Based Management of Inflammaging in CKD and Renal Replacement Therapies

**DOI:** 10.3390/nu13010267

**Published:** 2021-01-18

**Authors:** Vincenzo Losappio, Barbara Infante, Serena Leo, Dario Troise, Martina Calvaruso, Piercarla Vitale, Stefania Renzi, Giovanni Stallone, Giuseppe Castellano

**Affiliations:** Nephrology, Dialysis and Transplantation Unit, Department of Medical and Surgical Sciences, University of Foggia, Viale Pinto Luigi 251, 71100 Foggia, Italy; villy79@yahoo.it (V.L.); barbarainf@libero.it (B.I.); sereleo1989@libero.it (S.L.); dario.troise@gmail.com (D.T.); martinacalvaruso88@gmail.com (M.C.); piercarlavitale@libero.it (P.V.); renzi.stefania93@gmail.com (S.R.); giovanni.stallone@unifg.it (G.S.)

**Keywords:** end-stage renal disease, inflammaging, nutrition, microbiota, Mediterranean diet, dialysis, transplantation

## Abstract

Access to renal transplantation guarantees a substantial improvement in the clinical condition and quality of life (QoL) for end-stage renal disease (ESRD) patients. In recent years, a greater number of older patients starting renal replacement therapies (RRT) have shown the long-term impact of conservative therapies for advanced CKD and the consequences of the uremic milieu, with a frail clinical condition that impacts not only their survival but also limits their access to transplantation. This process, referred to as “inflammaging,” might be reversible with a tailored approach, such as RRT accompanied by specific nutritional support. In this review, we summarize the evidence demonstrating the presence of several proinflammatory substances in the Western diet (WD) and the positive effect of unprocessed food consumption and increased fruit and vegetable intake, suggesting a new approach to reduce inflammaging with the improvement of ESRD clinical status. We conclude that the Mediterranean diet (MD), because of its modulative effects on microbiota and its anti-inflammaging properties, may be a cornerstone in a more precise nutritional support for patients on the waiting list for kidney transplantation.

## 1. Introduction

Inflammation is a typical feature of end-stage renal disease (ESRD) and contributes to cardiovascular disease (CVD), protein-energy wasting (PEW) and premature death [1,2,3]. 

ERSD is linked to a chronic proinflammatory cytokine production and reduced clearance due to the progressive renal failure with retention of high molecular weight toxins and protein bund toxins (PBUT) that are responsible for oxidative damage. In patients on Renal Replacement Therapies (RRT), this inflammatory state is worsened by overhydration, poor dialyzer membrane biocompatibility, anticoagulation, vascular inflammation, and comorbidities such as diabetes, hypertension, obesity, malnutrition, and heart failure [4].

Recently, it has been recognized the possibility of a nutritional approach tailored to deal with this state of persistent, low-grade, chronic inflammation by reducing proinflammatory cytokines levels such as IL-1B, IL-6, IL-8, and IL-18 [5,6]. Therefore, the modulation of inflammaging processes might be at the center of a multidisciplinary scientific debate, with the primary objective to reduce red meat consumption and increase dietary fiber, leading to a modulation of macronutrients intake to improve the clinical conditions of patients in RRT waiting for kidney transplantation [7].

## 2. Epidemiology

According to USRDS, in 2017, almost the 50% of incident dialysis patients in the USA were older than 65 years. This trend is similar in all the western countries, with only 3–5% of patients accessing peritoneal dialysis (PD) and 1–2% receiving kidney transplantation [8]. Patients older than 65 years on dialysis have a higher mortality rate compared to younger patients, and their survival is conditioned by comorbidities such as vascular and cardiac disease. These conditions are tightly connected to ESRD and RRT and are related to unchangeable risk factors (age, sex, and cardiovascular disease familiarity) and changeable risk factors (lifestyle, hypertension, dyslipidemia, diabetes, and immunosuppressive drugs), defining a pattern of “Renal Frailty Phenotype” [9,10].

Tamura and colleagues evaluated elderly functional status before and after RRT, demonstrating a significant and permanent decline in patients’ functional status [11]. This impairment was also observed by van Loon, who assessed the clear relationship between malnutrition, frailty, and mortality and demonstrated the cognitive and functional impairment that led to increased mortality rate [12]. Interestingly, Dalton and colleagues identified a pattern of systemic inflammation in patients with serious psychological eating disorders related to anorexia nervosa, characterized by higher IL-6 serological levels compared to those detected in healthy controls [13]. Also, clinical depression is characterized by increased systemic inflammation, suggesting a possible influence on mental health and wellbeing [14]. These associations with poor outcomes have also been reported in other studies, suggesting the pattern of a multisystemic frailty condition [15,16]. 

On the contrary, kidney transplantation improves life expectancy, guarantees a survival advantage compared to other RRT, and has a lower financial burden compared to dialysis, offering a better quality of life (QoL) [17]. This is the reason why transplantation considered the gold standard for ESRD treatment [18,19]. Effectively, the mortality for kidney recipients is 68% lower than that for waiting list patients on RRT [18]. Gill and colleagues showed a 4-year life expectancy gain in non-elderly kidney transplant recipients when compared to waiting list patients [20], while recipients aged between 70–74 years old had an increased life expectancy of 1 year when compared to dialysis patients. 

Epidemiological data has also shown that a fast-growing portion of patients affected by ESRD on the waiting list is becoming older and frailer [21]. A great number of hemodialysis patients expire before kidney transplant [22], and RRT patients’ clinical conditions often worsen on the waiting list, making them unfit for transplantation if compared to non-frail patients [23]. Recent data showed a 44% prevalence of frailty in dialysis patients aged less than 40 years and a 78% in patients above 70 years, with an increased functional decline during the first 6 months of RRT in older patients [24]. Moreover, the incidence of frailty on waiting list patients ranges from 20% to 40% [25]. The absence of specific guidelines that define a fragility score threshold to exclude a patient from the waiting list leads to an underestimation of this problem and the consequent onset of post-transplant comorbidities, with increased graft failure and mortality.

Even for these patients, recent literature has shown good results in terms of QoL and survival after kidney transplantation [26,27], as well as in the setting of elderly recipients accepting suboptimal kidneys [28]. The European and American International Societies have consequently edited their guidelines for kidney transplantation without specific age contraindications [29], thus increasing the number of older patients on the waiting list. Therefore, the clinical monitoring of these changes [30] and their correction could be crucial to reduce the negative impact on graft and patient survival after transplantation [31].

## 3. Inflammaging in ESRD Patients

A first definition of “inflammaging” was developed by Franceschi and colleagues [32]. Starting from the “Disposable Soma Theory” [33] and according to the “Network Theory of Ageing” [34], they redefined the aging process, according to longevity models, as a “global reduction in the capability to handle with a variety of stressors and a concomitant progressive increase in the proinflammatory status,” thus including in a more comprehensive theory the previous MARS (Mitochondria, aberrant proteins, radicals, scavengers) model for intracellular damage and allowing the transition from “stochastic” toward “network” theories of aging.

In the setting, frailty could be considered the clinical manifestation of inflammaging, with a loss of resilience and reduced capacity to respond to health stressors [35]. In 2010, Brown and Johansson [36] suggested that frailty was a more powerful prognostic predictor than simple age due to its stronger correlation with 1-year mortality and hospitalizations rate. Inflammaging is common in the CKD population [37]. A cross-sectional analysis of the baseline data in the FEPOD (Frail Elderly Patient Outcomes on Dialysis) study showed that inflammaging is associated with worse QoL scores for patients on dialysis, independently of RRT modality [38]. Moreover, inflammaging is also associated with increased cognitive dysfunction [23] and mortality [39] and contributes to an increased vulnerability to external stressors in pre-dialysis and dialysis patients [10].

Behind clinical manifestations of frailty, the inflammaging process is characterized by biological alterations in the setting of CKD. Metabolic acidosis is responsible for a negative protein balance [40] that reduces protein synthesis and induces GH resistance. This is consequent to intracellular protein degradation mechanisms and to the upregulation of caspase-3 and the ubiquitin-proteasome system [41], the latter being related to a condition of insulin resistance that determines a decreased availability of phosphatidylinositol 3 kinase (PI3K) [42]. Also, TNF-α and IL-6, overexpressed in CKD patients, exert their catabolic effects with the stimulation of the ubiquitin proteasome complex and through the downregulation of anabolic pathways mediated by IGF and the dysregulation of the mTOR pathway [43]. Inflammatory processes are also implicated in autoimmune glomerulonephritis and transplant rejection, leading to progressive endothelial and tubular dysfunction [44,45]. 

Frail and uremic patients share increased apoptosis processes linked to the activation of NF-kB and Caspase 8, thus suggesting a process of accelerated aging [46] that is also sustained by Oxidative Stress (OS), a major contributor to biological aging. OS is particularly increased in ESRD [47] because of dysfunctional mitochondria, dysregulation of calcium, phosphate metabolism, and the mutation in Klotho genes [48], which are strongly linked to cancer development [49,50].

Damaged DNA and related epigenetic changes are also responsible for accelerated kidney senescence [51] in a vicious circle that makes cells more prone to uremic toxins and OS damage [52]. Recent literature has suggested a pivotal role for the Nuclear Factor Erythroid 2 Related factor (Nrf2). Patients with chronic inflammatory diseases such as CKD show reduced Nrf2 expression, which exerts cytoprotective effects against damage induced by ROS, upregulating an incredible range of genes with anti-inflammatory and antioxidative functions. This crosstalk between NF-kB and Nrf2 might led to improve effective anti-aging strategies [53]. 

Moreover, patients affected by ESRD or in RRT are subject to accelerated aging phenomena [54], with alterations displayed at the intracellular level and senescence of all physiological domains, particularly of the immune system. Through the evaluation of relative telomere length [RTL], Crepin and colleagues showed a comparable senescence status in 40-year-old uremic patients and 75-year-old healthy controls [55]. Uremic patients showed a reduction of circulating B cells, increased cellular apoptosis and progressive lymphopenia [56], and decreased number of plasmacytoid dendritic cells, natural killer (NK) cells and lymphoid T cells [57,58]. 

Because of inflammaging, B and T cells are premature not only by number but also by function, showing increased proapoptotic molecules that make cells more prone to death [59]. In addition, reduced circulating naive T-cells are not only responsible for less efficient immunosurveillance and, consequently, higher neoplastic risk, but their oligoclonal TCR profile is involved in a process of progressive loss of immunological specificity, making their function less efficient [60]. Low hormonal levels, malnutrition, decreased growth factors, and cytokines such as IL-2 or IL-7 [61,62,63] may contribute to this premature-aging phenotype. 

According to this new vision and the interplay between genetic and environmental components, nowadays we can clinically define inflammaging characterized by sarcopenia, vascular calcifications, cardiovascular hypertrophy. We can also biologically define inflammaging by premature immune senescence with consequent susceptibility to infections, malignancy, and chronic inflammation [51,64,65]. At the same time, a great interest has been developed about the interaction of this process with environment and nutrition [66], particularly for CKD and RRT patients.

## 4. Inflammaging, Western Diet and CKD/RRT Patients

Lesson learned from nonhuman primates reveal how the adoption of energy-dense food, as in the Western diet (WD), is not only related to the development of chronic diseases such as CVD, CKD, and diabetes [67], but also modifies the microbiome [68], increasing inflammation, cellular damage, and the risk of neoplasia. Colorectal cancer has a major diffusion in western countries because of the increased red meat consumption [69], and is a pathology of particular interest. Colorectal cancer is at the center of progressive microbiota modifications, including accelerated senescence mechanism due to inflammation, hyperphosphatemia [70], and progressive lack of anti-inflammatory pathways such as Nrf2 and its crosstalk with NF-kB [53].

Data collected from the USA Center for Disease Control and Prevention show a 32.4% prevalence of obesity with BMI higher than 30 compared to a 30.5% prevalence of BMI values associated with normal weight. This condition is evident worldwide [71], and is related to the availability of energy-dense food and connected to the rising prevalence of chronic diseases [72], with direct connection to a disproportionate red meat consumption [73]. This switch toward an imbalance diet, which lies on specific social and cultural environment, favors inflammaging. Moreover, social and economic factors seem to influence food availability, variety, and its protective CV effect: Higher educational qualification is associated with better competence in food choice. This could be also part of the high obesity rate observed in USA population. Large metropolitan centers, because of the demographic and social pressure, offer small housing with reduce or lacking cooking spaces. Food is chosen, not cooked, because of psychosocial and cultural practice, with preference for fast and tasty red meat. The unconscious development of chronic diseases is linked to the lack of antioxidant agents and impaired protective cellular mechanism [74,75].

Lifestyle factors such as sedentary attitude, reduced exercise and wrong alimentary regimens, as well as environmental factors may influence and modify inflammaging processes in patients on RRT with detrimental effects on the immune system and accelerated aging phenomena [76]. In the setting of dialyzed patients, a sedentary lifestyle with poor physical activity, due to fatigue on dialysis days or reduced available time, motivation, physical problems, and pain, is considered a modifiable risk factor and may influence cardiovascular risk [77]. Conversely, physical activity and exercise could reduce depressive symptoms and increase self-well-being, energy supply, and appetite [78].

**Figure 1:**
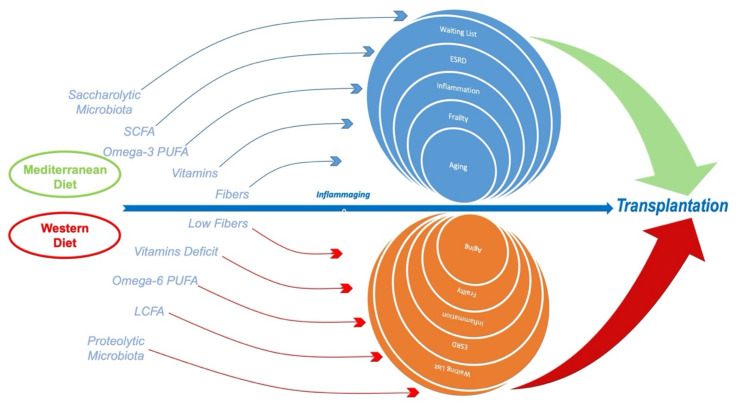
The Mediterranean diet, compared to Western diet, is based on the increased intake of long-chain fatty acids from fish and plant oils; fibers through legumes, fruits, and vegetables; and whole food grains. This approach may lead to better controls of inflammaging and malnutrition in end-stage renal disease (ESRD) with successful renal transplantation.

In addition, ESRD patients should control inorganic phosphate assumption, since it is often added to processed food and widely used for meat-derived preparations as a taste enhancer [79]. This phosphate is rapidly absorbable and more dangerous than the organic one. The necessity to control its assumption [80] is suggested among RRT patients and the general population [81,82]. In addition, animal-derived food presents high potassium content [83] and should be avoided in favor of plant-derived food, which has been reduced or banned in old nutritional programs [84]. The WD should also be avoided in RRT for the well-recognized proinflammatory effects related to its high sugar [85] and saturated fats intake [86], reduced content in fiber, complex carbohydrates, micronutrients, and lack of bioactive molecules such as omega-3 polyunsaturated fatty acids and polyphenols [87]. The increased red meat consumption in WD, especially the consumption of processed meat, leads to a microbiome imbalance toward a putrefactive profile and to increased phosphate assumption. In turn, this leads to alterations in Klotho genes that favor not only chronic inflammatory disease, but also progeria [48], probably through the downregulation of Nrf2 with consequent exposure to increased ROS damage [53] (Figure 1). 

Inflammation due to prolonged exposure to WD also determines an increased uptake of LPS, because of amplified gut leakiness interacting with TLR4 and innate immune system activation [88,89,90]. These alterations are consisted with reports suggesting higher colorectal cancer risk for red and processed meat consumer [91] compared to the protective effects related to higher doses of vegetal proteins [92].

## 5. Microbiome, Inflammaging and CKD/RRT Patients

In CKD patients, the large intestine assumes an excretive role to remove uremic toxins and preserve noble molecules, acting as a compensatory mechanism for nephron failure [93]. This massive uremic load affects colonic bacterial environment [94], worsening the uremic state until the complete loss of renal excretion function and the consequent block of their clearance [95]. At the same time, CKD patients present higher levels of C Reactive Protein (CRP), IL-6, and TNF-α compared to healthy controls [96,97], which are related to uremic toxins retention linked to intestinal dysbiosis [63].

This chronic inflammatory condition is characterized by a progressive alteration of the resident microbes of the gut, the microbiota, and by its gene heritage and coding capacity connected to the environment, the microbiome, exerting a pivotal role promoting or sustaining CVD, inflammatory bowel disease (IBD), diabetes, obesity, cancer, and malnutrition [98]. 

The gut microbiota is a dynamic supplementary organ, residing in the large intestine. The gut microbiota is metabolically active, in the middle of a dense network with the kidney, heart, immune system and many other organs [99]. It affects general health status, exerting protective and trophic functions influencing local and systemic metabolism and immunity [100,101]. Its microbial community includes about 1014 bacterial cells, located mostly in the colon, and represents the widest container of no self-antigens in human body [102]. It takes part in realizing digestion, facilitating complex carbohydrates adsorption, and preserving micronutrient homeostasis, such as amino acids and vitamins [103]. According to microbiota composition, microbiome exerts a pivotal role preserving immune homeostasis and enhancing anti-inflammatory conditions. In particular, the gastrointestinal apparat seems to attend immune tolerance aside from reacting to pathogenic stimuli [104].

The microbiome controls the metabolism and immunological network via two main catabolic pathways: The saccharolytic way, where bacteria fermenting carbohydrates work, and the proteolytic way, involving bacteria dominantly fermenting proteins. When species like Clostridium, Bacteroides, and Enterobacterium prevail, gut microbiota plays out protein fermentation or “putrefaction” with increased production of urea, ammonia, indoles, phenols, and other microbial uremic toxins as catabolic end products, thus switching microbiome potential to a proinflammatory profile [105]. Increased red meat consumption and lack of traditional food observed in WD perfectly fits this outline [98], impairing oxidant defense in mammalian cells [106].

On the contrary, protective symbiotic colonic microbes, primarily Bifidobacterium and Lactobacilli, execute saccharolytic fermentation, converting polysaccharides in monomeric sugars which are finally hydrolyzed in short-chain fatty acids (SCFA) [107]. Acetate, butyrate, and propionate are saturated SCFA, produced through dietary fiber fermentation by anaerobic colonic bacteria. In experimental allergic inflammation, mice were protected when fed with high-fiber diets, showing increased levels of SCFAs bound to GPR41 and GPR43 receptors [108]. In particular, Butyrate displays immune-modulating and anti-inflammatory competences [109] through the production of transforming grow factor-beta (TGF- β) and the ability to lower proinflammatory cytokines, such as IL-6, IL-17 and interferon-γ (INF-γ) [110,111]. Through the “microbe-associated molecular patterns (MAMPs)” production, the saccharolytic pathway promotes the enterocyte secretion of TGF- β, Il-25, and IL-33, which preserve the intestinal epithelial barrier and the tolerogenic environment [110]. TGF-β stimulates T-reg cells proliferation [112], and the bacteria component LPS increases forkhead box p3 (Foxp3) T-reg cells in mesenteric lymph nodes [113]. The polysaccharide A from Bacteroides Fragilis influences Th1-Th2 interplay, activating CD4+ cells [114]. In turn, CD4+ cells become Foxp3 T-reg cells, an important source of IL-10, an anti-inflammatory cytokine [115].

To understand the relationship between metabolic diseases and its effects on microbiome, several studies have investigated CKD-related gut dysbiosis and its link with diet [116] according to the strong relationship between humans, the environment, and illness [98]. RRT patients follow a strict dietetic regimen where fruits, vegetables and high-fiber foods are often reduced or forbidden, and protein products are the most available food [117]. This dietary restriction induces changes in microbiota composition, and the consequent microbiome switch from a saccharolytic to proteolytic profile, thus enhancing uremic toxin production, mainly urea, ammonia, p-cresyl sulfate (PCS), and indoxyl sulphate (IS) [118]. Many authors have described the effects of PCS, IS, and other uremic toxins against the gut epithelial barrier itself: They cause derangement of the epithelial tight junctions up to the loss of its integrity [119], which is important for gut activities [120]. When the gut barrier is opened, mucosal antigens are exposed and bacteria can translocate to the blood circulation, resulting in endotoxemia, consequent immunological impairment, and systemic inflammation [121]. Moreover, uremic molecules increase pathological reactive oxygen species (ROS) production, aberrant cellular proliferation, and cellular senescence with the secretion of proinflammatory cytokines [122].

## 6. Nutritional Intervention Against Inflammaging in CKD/RRT Patients

Given the adequate depurative dose by RRT, patients, particularly those on the waiting list for kidney transplantation, should modulate their quantity and quality of food [123]. To avoid malnutrition, in ESRD and hemodialysis patients, a general energy intake of 30 Kcal/Kg/day with a 1 g/Kg/day daily protein assumption is suggested. This intake should be maintained in the first months after transplantation and then reduced to 0.8 g/Kg/day in transplant recipients with adequate graft function. Protein and energy intake should be always adjusted for gender, age, and levels of physical activity according to international indications [124], ensuring caloric supplementation during the dialysis session [125] or acute kidney injury [126]. Oral nutritional supplements, with no added phosphate, should be used for short periods to satisfy specific clinical needs, with a balanced composition of macronutrients tailored for patients’ necessities [127,128].

According to recent nutritional evidence, dietary habits should be more focused on avoiding processed foods than on limiting the consumption of plant-derived aliments [7,129] (Figure 1). Higher consumption of plant-based proteins should be suggested for all CKD stages to reduce the burden of inorganic phosphate [130]. This nutritional approach is “desirable” considering the burden of potassium, phosphate, and chemical products, such as taste enhancers and chemical colorants daily consumed for the large consumption of industrial-processed foods [131]. Adequate nutrient intake is possible for the availability of new and more efficient drugs and RRT tailored for potassium and phosphate removal and intake [84].

According to the general view, the potassium content in fruit and vegetables discourages its implementation because of the risk hyperkaliemia. However, recent literature [84] sustains a different approach according to the efficiency of new dialytic techniques. According to the American Kidney Fund (https://www.kidneyfund.org/assets/pdf/training/potassium-and-kidney-disease.pdf), The daily assumption of potassium for patients under RRT should be around 2500 mg. Interestingly, it is possible to observe that a 150 g serving of apple or pear contains less than 200 mg of potassium, a profile shared with other fruits such as strawberries, oranges, and pineapple. Moreover, vegetables with higher fiber content have 280 mg of potassium per 100 g serving. In addition, the potassium content of medium fish is less than the potassium estimated for beef, sheep, chicken, or turkey breast (higher than 350 mg for 100 g).

Moreover, less restrictions and more individualized approaches in ESRD hold beneficial effects compared to dietary restrictions [84]. Selective diets decrease patient’s QoL and could be harmful, because vegetable, fruit, and legume consumption is associated with higher fiber intake and its beneficial effect on gut microbiota and peristalsis, with reduced production of proinflammatory cytokines [132]. For this reason, if possible, even for RRT patients, a daily intake of six portions of fruit and vegetables is suggested [132]. Interestingly, 97% of hemodialysis patients have a daily consumption of dietary fiber lower than 25 g [133] and a reduced fiber intake comparable to healthy population [134]. This scenario should be taken into consideration, since the importance of dietary fiber was underscored in the 1999–2000 NHANES study through the evaluation of 4900 subjects. The subjects in the third- and fourth-highest quartiles of fiber consumption had a lower risk of elevated CPR compared to the other quartiles [135]. 

In addition, fibers decrease the serum levels of creatinine and urea [136] and the plasma concentration of several protein-bound gut-derived uremic toxins, such as p-cresyl-sulfate and indoxyl-sulfate, which are not efficiently removed by RRT [137] and are related to poor outcomes because of their accumulation [138]. In addition, fiber supplementation showed positive effect on mortality in CKD patients [134], but its optimal intake in this population has not been defined [136]. Therefore, there is consensus that the dietary fiber intake CKD/ESRD patients should be comparable to healthy population, about 25 g/day.

## 7. Mediterranean Diet, Inflammaging and CKD/RRT Patients

The recently published 2020 Nutritional KDIGO guidelines provide low-grade nutritional indications considering calories and protein intake and opinion-based nutritional indications for micronutrients and protein origins [139]. However, adherence to healthy diet was recently associated with lower risk for CKD progression and all-cause mortality [140]. In addition, adherence to the Mediterranean diet (MD) was associated with better graft function in kidney transplant recipients [141].

MD is characterized by a substantial intake of long-chain fatty acids derived from plant oils and fish, fibers through legumes, vegetables, fruits, and whole grains. This diet leads to reduced dyslipidemia and protection against lipid peroxidation and inflammation. This diet can also increase, when necessary, the dietary caloric load to control malnutrition by setting patients in a satisfactory nutritional status [142] (Figure 1). Fiber, abundantly present in fruits, vegetables, and legumes which are widely consumed in MD, is related to better glycemic control, as well as antioxidant and anti-inflammatory effects. At the same time, MD leads to higher concentration of adiponectin, which is known for its anti-inflammatory properties, with a potential shift and improvement of the intestinal microbiota. MD is also associated with the reduced risk of chronic disease such as CVD, cancer, and neurological diseases such as Alzheimer [143]. Recent literature has shown only a substantial role for typical nutrients introduced with MD and their effect on healthy ageing, but also the use of simple cooking techniques to reduce the exposure to polycyclic aromatic carcinogens [144].

**Figure 2:**
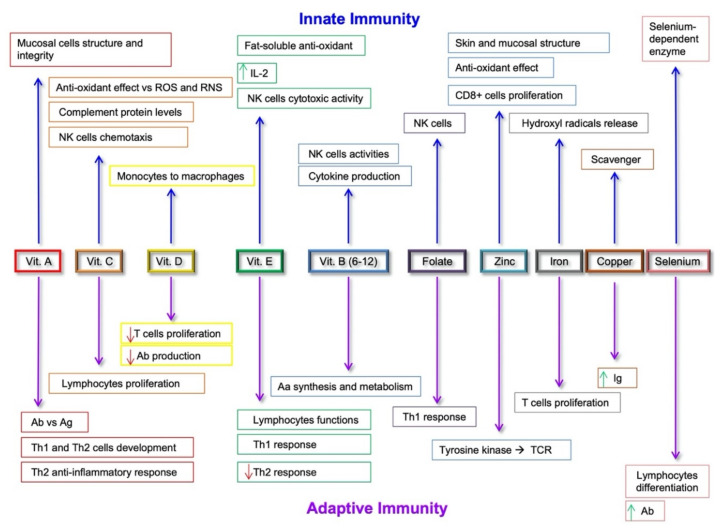
Effects of micronutrients on the modulation of innate and adaptive immunity systems. Abbreviations: ROS, Reactive Oxygen Species; RNS, Reactive Nitrogen Species; NK, Natural Killer; Ab, Antibodies; Ag, Antigens; Aa, Amino Acids; Ig, Immunoglobulins; TCR, T Cell Receptor. Green up arrows: upregulation; Red down arrows: down-regulation.

Omega-3 polyunsaturated fatty acids (ω-3 PUFAs) in olive oil and fish, abundant in MD, are essential fatty acids notoriously known for their protective and anti-inflammatory properties against CVD [145,146] (Figure 2). Studies in healthy elderly patients have suggested that ω-3 PUFAs also hinder anabolic resistance and sarcopenia and tickle muscle protein synthesis [147]. Compared to general population, RRT patients have reduced serological levels of ω-3 PUFAs, which are probably linked to a reduced intake of fish [146]. In 110 hemodialysis patients, after 12 weeks of ω-3 PUFAs and ω-3 PUFAs + vitamin E supplementation, there was a significant improvement in the subjective global assessment score (SGA-score) and other metabolic parameters improved [148]. Likewise, a 4-month supplementation of ω-3 PUFAs to RRT patients improved inflammatory markers, such as C-reactive protein, IL-6, IL-10, and TNF-α, with no effect on nutritional status markers such as bodyweight, albumin, pre-albumin, and transferrin [149].

Long-chain ω-3 PUFA exerts their anti-inflammatory effects by inhibiting TLR expression and by reducing proinflammatory cytokine production, with a specific effect also on TLR4 [150]. Increased consumption of fish products and reduced intake of vegetable oils improves the ω-3/ω-6 ratio, thus controlling the balance between anti-inflammatory and proinflammatory processes. The ω-3 anti-inflammatory profile depends on its ability to replace ω-6 in several metabolic pathways, thus reducing the levels of proinflammatory mediators [151]. ω-3 PUFA modulate proinflammatory gene expression through the inhibition of nuclear factor-kB (NF-kB) activity, and through the reduced expression of intracellular adhesion molecule (ICAM)-1, vascular cell adhesion molecule (VCAM)-1, and E-selectin [152] (Figure 2). If ω-6-rich diets increase the risk for inflammatory diseases, ω-3 consumption, instead, decreases this risk related to cancer development [153]. In vivo, dietary ω-3 PUFAs also exert anti-inflammatory effects via the inhibition of T cell proliferation and the production of IFN- γ and IL-17 [154] (Figure 2). Evidence from in vitro and animal models has shown the ability of dietary ω-3 PUFAs to increase pro-resolving functions of neutrophils and re-establish balanced innate immune responses [155].

MD also provides an adequate intake of micronutrients. Through a food frequency questionnaire, HD patients also showed a reduced intake of vitamin C, vitamin D, magnesium, zinc, lycopene, kryptoxanthin, and lutein [156]. Vitamin D, magnesium, and zinc also have anti-inflammatory effects [157,158] (Figure 2). Vitamin D, whose activation is impaired with progressive kidney failure, modulates the immune system through VDR receptors [157], while magnesium, which is stored in advanced CKD, reduces inflammation and is inversely associated with CRP levels [159]. Zinc, instead, acts as cofactor for enzymatic activities and influences T lymphocytes’ function. In addition, zinc, manganese, selenium, and copper deficiencies may determine impaired intracellular glutathione peroxidase function with its antioxidant. Because of their efficacy to neutralize free radicals, they should be monitored in restrictive, low-calorie diets and in patients in ESRD [156]. Dietary polyphenols, bioactive compounds found in vegetables and fruits, have shown a role in the regulation of inflammation [160] and have proven immunomodulatory and anti-inflammatory effects, lowering the risk for the development of CVD, cancer, and neurological diseases [161] (Figure 2).

MD could also positively impact the microbiome. Considering its plasticity and the nutrition capacity to dysregulate intestinal bacterial composition and physiological functions, dietary adjustments could be considered therapeutic interventions in these pathological conditions [162]. Nutritional strategies directly targeting gut microbiota might represent a correct way to restore human-microbiome relationship [163]. Selecting saccharolytic over proteolytic microbial colonies using dietary intervention represents the best method to directly modulate intestinal bacteria proliferation by blocking colonic adsorption of uremic toxins and subsequently decreasing systemic inflammation [164]. A large body of evidence has shown that plant-derived fibers, legumes, and unrefined cereals benefit intestinal health and improve microbiota metabolism and composition, stimulating the saccharolytic pathway and releasing SCFA [165]. 

In addition, increasing attention is now given to prebiotics, probiotics, and symbiotics [166]. Prebiotics are nondigestible substances contained in aliments, as well as water-soluble fibers, including beta-glucans, oligosaccharides, and soy oligosaccharides. These fermentation substrates ease the probiotics, the “good” intestinal bacteria, favoring their proliferation and working [167]. For this reason, World Health Organization has defined a probiotic as a “live microorganism which when administered in adequate amounts confer a health benefit on the host.” Symbiotics represent prebiotics and probiotics working synergically to ameliorate the probiotics’ environment and to provide adequate substrates to resident microbiome. Several researches have shown the capability of prebiotics, probiotics and symbiotics to remove uremic toxins and, consequently, to keep inflammation cascade, immune system impairment, disease progression, and related comorbidities under control [168].

## 8. Conclusions

Patients in ESRD or RRT are often characterized by malnutrition and frailty that are clinical manifestations of inflammaging. In this contest, a dietary approach based on MD, according to its immunomodulatory properties and antioxidant abundance, might prevent its detrimental effects.

A recent multinational cohort study conducted for 3 years involving over 8000 patients showed that compared to general population’s recommendations, HD patient’s intake of fruit and vegetable was consistently low. At the same time, a higher consumption was associated with all-cause mortality and non-cardiovascular death. Particularly, the higher consumption of fruit and vegetables was observed in Italy with a median of 12 serving per week [169]. These data are consistent with the Moli-sani study report on adherence to MD and cardiovascular protection in general population [170].

For this reason, the ongoing Kidney Disease Outcomes Quality Initiative (KDOQI) guideline recommends that every patient and/or caretaker should receive nutrition counseling when starting RRT. The choice of a nutrition able to fulfill these indications and support immune response is referred to as immune-nutrition, and different approaches have been studied to understand its possible effects [171]. In this effort, a tailored nutritional support, if associated with physical exercise [172], could guarantee a good nutritional status for ESRD patients and possibly reverse the clinical signs of frailty and musculoskeletal senescence [173]. 

Nutritional counseling should embrace not only the quantity and quality of food but also the environmental and social behaviors of patients with kidney disease. A proactive attitude against inflammaging, particularly in kidney transplant recipient, should be considered a necessary challenge to counteract CKD progression and preserve graft function. In this scenario, MD could represent a model to refer to for personalized nutritional programs in CKD/RRT patients in order to improve clinical conditions and outcomes in time for this particular population of patients.
